# Ubiquitylomics: An Emerging Approach for Profiling Protein Ubiquitylation in Skeletal Muscle

**DOI:** 10.1002/jcsm.13601

**Published:** 2024-09-16

**Authors:** Samuel O. Lord, Harvey E. Johnston, Rahul S. Samant, Yu‐Chiang Lai

**Affiliations:** ^1^ School of Sport, Exercise and Rehabilitation Sciences University of Birmingham Birmingham UK; ^2^ Signalling Programme The Babraham Institute Cambridge UK; ^3^ MRC Versus Arthritis Centre for Musculoskeletal Ageing Research University of Birmingham Birmingham UK; ^4^ NIHR Birmingham Biomedical Research Centre Sarcopenia and Multimorbidity University of Birmingham Birmingham UK

**Keywords:** diGly, mass spectrometry, proteomics, skeletal muscle, ubiquitin

## Abstract

Skeletal muscle is a highly adaptable tissue, finely tuned by various physiological and pathological factors. Whilst the pivotal role of skeletal muscle in overall health is widely acknowledged, unravelling the underlying molecular mechanisms poses ongoing challenges. Protein ubiquitylation, a crucial post‐translational modification, is involved in regulating most biological processes. This widespread impact is achieved through a diverse set of enzymes capable of generating structurally and functionally distinct ubiquitin modifications on proteins. The complexity of protein ubiquitylation has presented significant challenges in not only identifying ubiquitylated proteins but also characterising their functional significance. Mass spectrometry enables in‐depth analysis of proteins and their post‐translational modification status, offering a powerful tool for studying protein ubiquitylation and its biological diversity: an approach termed ubiquitylomics. Ubiquitylomics has been employed to tackle different perspectives of ubiquitylation, including but not limited to global quantification of substrates and ubiquitin linkages, ubiquitin site recognition and crosstalk with other post‐translational modifications. As the field of mass spectrometry continues to evolve, the usage of ubiquitylomics has unravelled novel insights into the regulatory mechanisms of protein ubiquitylation governing biology. However, ubiquitylomics research has predominantly been conducted in cellular models, limiting our understanding of ubiquitin signalling events driving skeletal muscle biology. By integrating the intricate landscape of protein ubiquitylation with dynamic shifts in muscle physiology, ubiquitylomics promises to not only deepen our understanding of skeletal muscle biology but also lay the foundation for developing transformative muscle‐related therapeutics. This review aims to articulate how ubiquitylomics can be utilised by researchers to address different aspects of ubiquitylation signalling in skeletal muscle. We explore methods used in ubiquitylomics experiments, highlight relevant literature employing ubiquitylomics in the context of skeletal muscle and outline considerations for experimental design.

## 1 Introduction

Skeletal muscle makes up approximately 40% of an individual's body mass. It is essential not only for movement but also for whole body metabolism [1]. Unsurprisingly, loss of muscle mass and quality is detrimental to an individual's quality of life and increases mortality risk [2]. Many individuals will experience muscle impairments throughout their lifespan, whether through muscle disuse, ageing or diseased states such as cancer, sepsis, heart disease, neurodegeneration and chronic inflammation [3]. This presents a growing burden not only on healthy living standards but also on healthcare systems, with muscle wasting costing the United Kingdom in excess of £2.5 billion per year [4]. Therefore, the development of pharmaceuticals that can help prevent or combat muscle diseases is an area of unmet clinical need. However, this goal requires a comprehensive understanding of the molecular mechanisms driving fundamental biological processes essential for maintaining healthy muscle function.

Skeletal muscle function is regulated by many biological processes, including mechanical signal transduction, energy metabolism and protein turnover. These processes are tightly regulated by signalling events that can switch specific pathways ‘on’ or ‘off’. Post‐translational modifications (PTMs) are often the drivers of such signalling events and are crucial for skeletal muscle function. One of these PTMs, protein ubiquitylation, is well known for its role in protein degradation [5], a process that regulates both skeletal muscle mass and protein quality. There is now overwhelming evidence that protein ubiquitylation also regulates a multitude of biological processes beyond degradation, regulating almost all aspects of cellular function and homeostasis [6].

**FIGURE 1:**
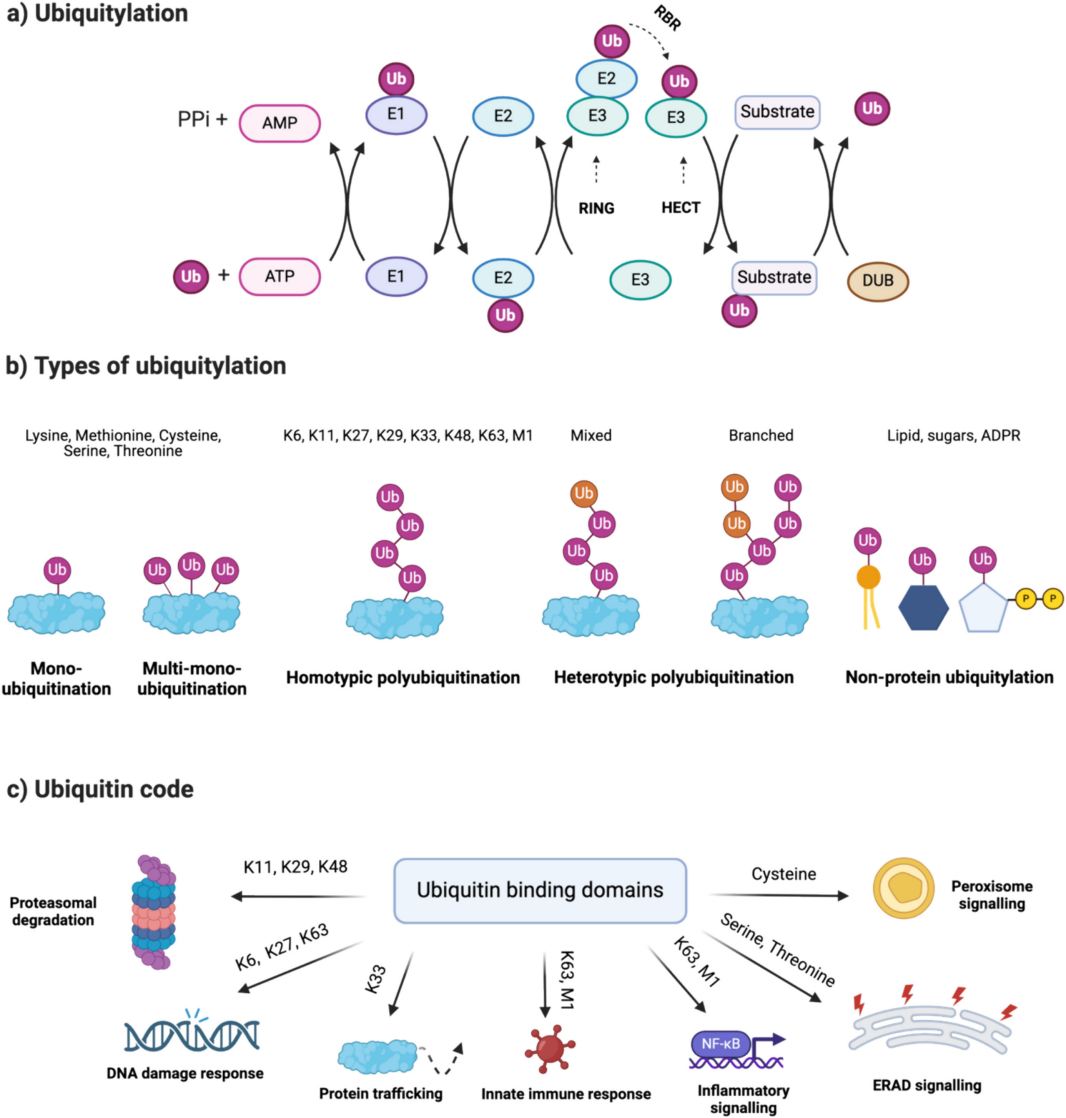
(a) Enzymatic cascade of reactions that occur during ubiquitylation. E3 ligases can be divided into three families: RING‐finger, HECT, and Ring‐Between‐Ring (RBR). Each have unique capabilities for transferring ubiquitin to the substrate. (b) The versatility of ubiquitylation, from different linkage sites (Lysine, Methionine, Cysteine, Serine, Threonine), chain topology (homotypic and heterotypic) and substrates (protein and non‐protein). (c) The ubiquitin code that uses ubiquitin binding domains (known as readers) to recognise specific ubiquitin‐linkage types, which signal for different biological processes in cells.

Protein ubiquitylation involves the covalent attachment of ubiquitin—a 76 amino acid protein—via its C‐terminal, predominantly onto internal lysine residues of target proteins (although the presence of N‐terminal protein ubiquitylation is also well established). Recent work has discovered that ubiquitin can also target internal cysteine, serine and threonine sites, or even non‐protein molecules [7, 8]. Protein ubiquitylation requires sequential ATP‐driven enzymatic reactions, involving ubiquitin‐activating (E1), ubiquitin‐conjugating (E2) and ubiquitin‐ligating (E3) enzymes (Figure 1a). Protein ubiquitylation can also be removed by deubiquitylases (DUBs). At present count, there are two E1s, nearly 40 E2s, more than 600 E3s and approximately 100 DUBs encoded in the human genome [9]. Proteins can be ubiquitylated in many ways: on a single amino acid by a single ubiquitin moiety (monoubiquitylation), on multiple amino acid residues by single ubiquitin moieties (multi‐monoubiquitylation) or on single or multiple amino acids by covalently linked ubiquitin chains (polyubiquitylation). Polyubiquitin chains exhibit distinct topologies, determined by the internal lysine (K6, K11, K27, K29, K33, K48, K63) or N‐terminal methionine (M1) used for chain elongation [10] (Figure 1b).

Characterising the nature of ubiquitin modification is an important step in determining its physiological consequences. The recognition of ubiquitylation topology is mediated by ubiquitin binding domains. Proteins equipped with these ubiquitin binding domains, often referred to as ‘readers’, play a pivotal role in transducing the ubiquitin signal into downstream outputs [11] (Figure 1c). Ubiquitylation can signal for various biological processes; therefore, ubiquitin binding domains often appear to be linkage‐specific, contributing to the complex network of ubiquitin signalling known as the ubiquitin code [12]. For instance, K48‐linked ubiquitin chains signal for proteasomal degradation, a process not generally attributed to homotypic K63‐linked ubiquitin chains [13]. Instead, K63‐linked ubiquitylation has a major role in diverse signalling responses including autophagy, endocytosis, DNA damage and immune response [14]. The role of other lysine‐linked ubiquitin chains are less well characterised but have roles in autophagy, cell cycle, DNA damage, immunity, degradation and protein trafficking [15]. M1‐linked ubiquitin chains (linear ubiquitylation) are heavily involved in inflammatory signalling through NF‐kB pathway [16]. The role of other non‐lysine ubiquitylation (serine, threonine or cysteine) is still an emerging field, with a role in endoplasmic reticulum‐associated degradation (ERAD) and beyond [7, 17]. However, it is essential to note that our current understanding of the ubiquitin code is derived from cellular models; the extent to which the same diversity exists in skeletal muscle requires further investigation.

## 2 Methods for Studying Protein Ubiquitylation

Defining ubiquitin‐mediated signalling networks is complicated by several sources of difficulty in capturing ubiquitylation events. Ubiquitylation can be a very transient modification due to the removal of ubiquitin linkages by DUBs. Recent work has shown that DUBs regulate at least 40000 unique sites on ubiquitylated proteins [18]. In addition, given that protein ubiquitylation is widely used to target proteins for degradation, the window for detecting such proteins is reduced by their high turnover rates. In human cell lines, the median half‐life of global ubiquitylation sites is 12 min, which is substantially shorter than the bulk of the cellular protein repertoire, where over 95% have a half‐life greater than 8 h [19, 20]. As a consequence, ubiquitylated proteins have a low stoichiometry when compared to non‐ubiquitylated proteins.

For this reason, preserving protein ubiquitylation at the point of sample collection is an essential step in obtaining a robust readout. DUBs display promiscuous activity when released in tissue or cell homogenates. Including DUB inhibitors such as EDTA/EGTA (inhibit metallo‐proteinases) and 2‐chloroacetamide/Iodoacetamide/N‐ethylmaleimide/PR‐619 (inhibit cysteine proteinases) in the lysis buffer ensures that ubiquitylated proteins are kept as they were in the intact cell or tissue. Unlike the addition of protease inhibitors, it is not standard practice to include DUB inhibitors in lysis buffers, at least not at the recommended concentrations [21]. Including sufficient concentrations of DUB inhibitors is particularly important when using non‐denaturing lysis buffers and when handling samples outside of low temperatures. To try and capture degradation‐borne proteins, proteasome inhibitors such as bortezomib and MG‐132 are often added into cells for a short period prior to lysis. However, due to the damaging consequences of preventing protein degradation, proteasome inhibitors are less suitable for in vivo studies. Furthermore, proteasome inhibitors display off‐target effects including an increase in compensatory degradation pathways, such as autophagy [22], and a decrease in non‐degradative ubiquitylation signals, such as histone ubiquitylation [23].

Considerations should be made for the choice of sample preparation. Sample preparation should ensure the proteins of interest are solubilised and retained for analysis. Certain proteins can be difficult to analyse due to their intrinsic properties. For example, transmembrane proteins are more hydrophobic than globular proteins [24]. Protocols have been developed to ensure optimal extraction and recovery of membrane‐bound proteins [25, 26]. Capturing such proteins may be desirable as ubiquitylation plays a major role in the secretory pathway and plasma membrane protein transport [27]. Therefore, the chosen method of sample preparation should be tailored towards the type of proteins at the focus of each study.

To date, most studies have relied upon antibody‐based detection (e.g., western blotting) to investigate protein ubiquitylation in skeletal muscle. Whilst these approaches can provide semi‐quantitative analysis of ubiquitylation, they do have limitations. A notable limitation is antibody availability; for instance, not every ubiquitin linkage type has a commercially available antibody and therefore cannot be detected by immunodetection assays (e.g., western blotting, immuno‐cytochemistry/histochemistry) [28]. Commercialised ubiquitin‐linkage‐specific antibodies were first developed for K48‐ and K63‐linked ubiquitin chains [29], which is a major contributing factor towards why these ubiquitin chains are best studied and comparatively better understood than other chain types. To our knowledge, in contrast with much smaller PTMs such as protein phosphorylation, there are very few commercially available antibodies that allow detection of a specific protein only when it is ubiquitylated at a given amino acid. Therefore, to determine whether a protein has been ubiquitylated, one must first include an enrichment step to selectively capture ubiquitylated proteins before blotting for the protein of interest, or vice versa.

Most commonly, ubiquitin enrichment from biological material involves the use of ubiquitin binding domains from various proteins that evolved to recognise ubiquitin signals (e.g., the proteasome shuttle‐factors Dsk2 from yeast *S. cerevisiae* or UBQLN1 from humans). Through the recombinant expression of ubiquitin binding domains with different intrinsic binding specificities and/or the fusion of multiple ubiquitin binding domains into repeating units, for example, MultiDsk [30], tandem ubiquitin binding entities (TUBEs) [31] and OtUBD [32], researchers have successfully generated reagents with enhanced and selective affinity towards monoubiquitin or polyubiquitin chains of different lengths and/or linkages [21]. Even after successful enrichment, researchers may be forced to focus on proteins with the most specific and sensitive antibodies available for detection. Subsequently, many proteins become neglected simply because they are more difficult to investigate, which slows down progress in the field, or at the very least, builds an incomplete model.

Tackling many of these issues, mass spectrometry has emerged as a powerful tool for in‐depth analysis of protein ubiquitylation (‘ubiquitylomics’). Mass spectrometry has the potential to identify thousands of ubiquitylated proteins and provide the ubiquitylation site on each peptide. Accordingly, mass spectrometry is responsible for the detection of many novel ubiquitylated substrates and sites that were previously limited by the detection tools available [33]. Furthermore, protein groups and biological pathways enriched in ubiquitylomics datasets can be identified through computational tools such as gene set enrichment analysis (GSEA) [34]. Such findings create new research avenues to explore previously neglected roles of protein ubiquitylation.

The aim of this review is to articulate how ubiquitylomics can be utilised to study protein ubiquitylation events in skeletal muscle. We first highlight methodological approaches available within ubiquitylomics workflows and their applications for understanding biology and developing drug targets. Next, we review studies that employ ubiquitylomics to advance our molecular understanding of muscle biology. Finally, we discuss the challenges associated with performing ubiquitylomics in skeletal muscle and outline key considerations for experimental design.

## 3 Mass Spectrometry–Based Ubiquitylomics

Mass spectrometry is a highly sensitive tool for detecting whether a protein has been ubiquitylated, simply by a change in peptide mass. This most commonly involves bottom‐up proteomics, in which proteins are first cleaved into smaller peptides at the carboxyl side of arginine and lysine residues by trypsin. Following trypsin digestion, peptides that have been ubiquitylated will retain a double glycine remnant (‘diGly’) protruding from the site of ubiquitylation. The diGly remnant adds 114.04 Da to the peptide mass, distinguishing itself from the unmodified parent peptide. This mass shift was first utilised in 2003 to identify 110 ubiquitylation sites on 72 proteins [35]. Due to the low stoichiometry of protein ubiquitylation, this study along with those conducted over the next several years relied on non‐native expression of tagged ubiquitin to selectively enrich ubiquitylated proteins. Whilst ground‐breaking, these early studies suffered from high protein background and were limited to cell‐based experiments where non‐native ubiquitin could be expressed. Over the last decade, considerable developments at the level of biochemical ubiquitin tools, mass spectrometric instrumentation and computational methodologies have spurred a growing interest in the use of ubiquitylomics.

### 3.1 Ubiquitin Remnant Peptide Enrichment

Perhaps the most consequential development that revolutionised the ubiquitylomics field came in 2010, allowing ubiquitylated sites to be selectively enriched using an antibody [33]. Since most protein ubiquitylation occurs on lysine residues, this antibody was developed to recognise the lysine epsilon diGly (K‐ε‐GG) ubiquitin remnant motif. This antibody was developed for peptide immunoprecipitation, that is, to selectively enrich ubiquitylated peptides after trypsin digestion, prior to mass spectrometry‐based bottom‐up proteomics. A year later, this antibody enrichment platform was used to recognise over 10 000 ubiquitylation sites on ~5000 proteins in human cell lines [13, 23]. These studies illustrated the tremendous potential of mass spectrometry for large scale analysis of protein ubiquitylation. Various protocols have since been developed to improve the efficiency and versatility of K‐ε‐GG antibody enrichment, including chemical cross‐linking to the beads [36], on‐antibody TMT labelling [37], magnetic beads and automated processing [38].

Since the development of the K‐ε‐GG antibody, a few other antibodies have been developed to enrich different ubiquitin remnants on peptides. One group has developed an antibody termed UbiSite that has greater specificity for ubiquitylated peptides over similar peptides with ‘ubiquitin‐like’ (UBL) PTMs [39]. This antibody recognises a larger 13‐residue remnant (after Lys‐63 in the ubiquitin sequence) which is brought about following digestion with LysC instead of trypsin. This reliance on a larger ubiquitin remnant epitope presents two main advantages to UbiSite when compared with K‐ε‐GG: (1) UbiSite recognises a broader range of ubiquitylation sites, not just those formed on a lysine residue, and (2) UbiSite does not recognise other UBL PTMs, such as NEDDylation and ISGylation, both of which are indiscriminately enriched by the K‐ε‐GG antibody. The second point is likely more important when dealing with UBL‐activating conditions such as inflammation, as whilst ubiquitin makes up ~95% of total ubiquitin and UBL signal in resting cells, ISGylation significantly increases under interferon stimulation [13]. More recently, another group has developed an antibody specifically targeting diGly remnants on the N‐terminus of peptides, corresponding to N‐terminus ubiquitylation [40]. Together, these ubiquitin remnant motif antibody enrichment tools have overcome barriers previously encountered in ubiquitylomics analysis.

### 3.2 Ubiquitin Chain Analysis

**FIGURE 2:**
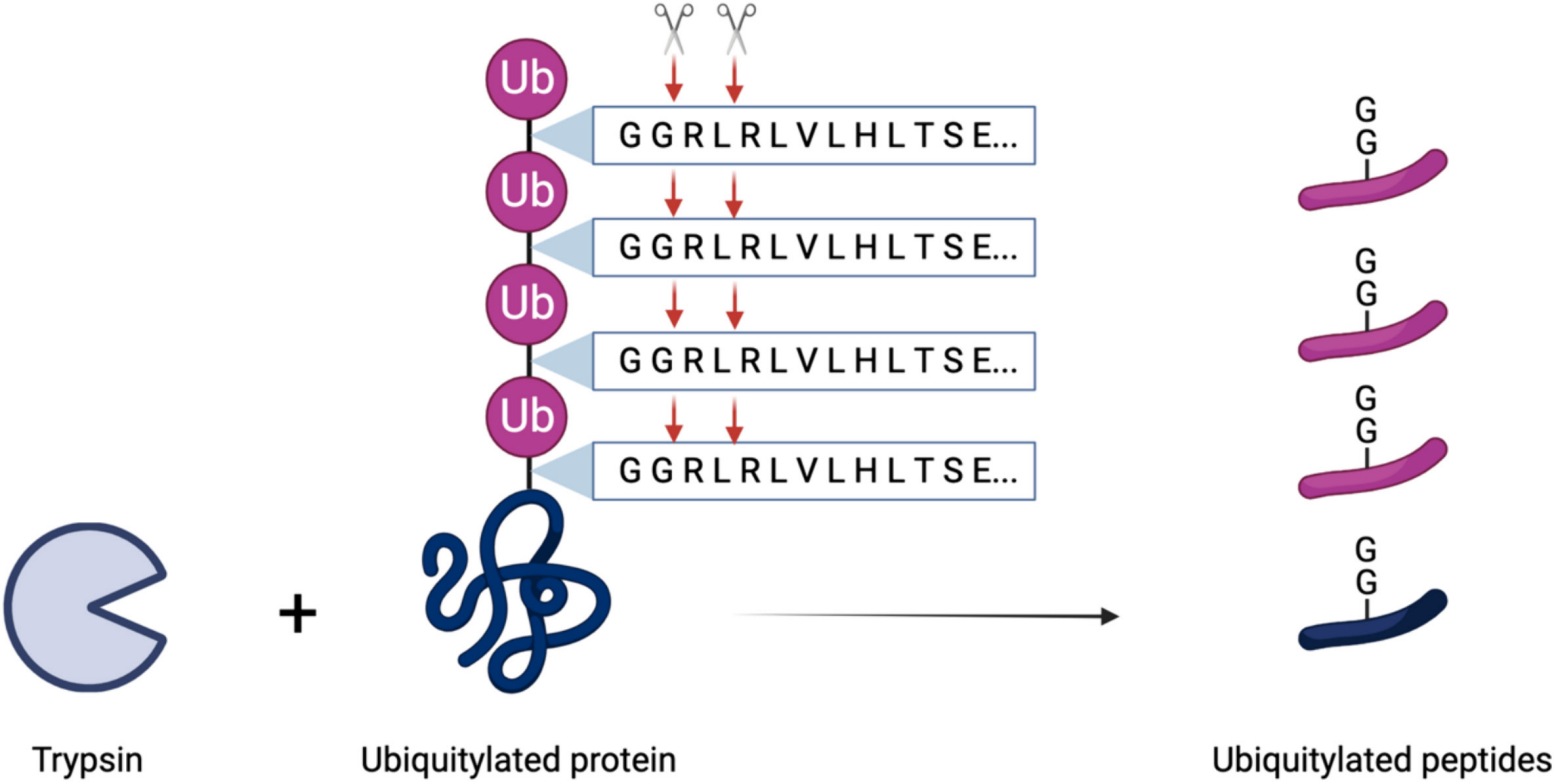
Digestion of ubiquitylated proteins. Trypsin cleaves at the C‐terminal side of Arginine (R) and Lysine (K) residues, which are found throughout the ubiquitin sequence. During trypsin treatment, ubiquitin chains are therefore digested into multiple peptides. Digested ubiquitin chains can be identified by the signature double glycine (GG) remnant on ubiquitin peptides; however, the substrate is no longer attached and cannot be identified in a complex mixture.

A major limitation with the bottom‐up proteomics approach, such as those used for ubiquitin remnant peptide enrichments, is the loss of ubiquitin architecture following the digestion of proteins into peptides. The enzymatic cleavage disassembles ubiquitin linkages, preventing the determination of ubiquitin chain topology on identified substrates (Figure 2). This complicates the inference of the biological impact associated with each ubiquitylation site, given that the ubiquitin chain determines its function (Figure 1c). Alternative methods must be considered if obtaining information of ubiquitin chain types is paramount.

One strategy is to take advantage of chain‐specific TUBEs, affimers or antibodies to selectively enrich for proteins with specific ubiquitin chain types prior to mass spectrometry [41]. These enrichment techniques enable the acquisition of chain‐type linkage information for each protein. However, ubiquitin chain enrichment tools can suffer from cross‐reactivity and require larger amounts of starting material. In addition, it is important to note that protein‐level enrichments are less suitable for identifying ubiquitylation sites, given their low stoichiometry in relation to non‐ubiquitylated sites within a protein's amino acid sequence. Additional methods for assessing ubiquitin chain topology include Ub‐clipping and/or middle‐down mass spectrometry to retain some of the ubiquitin chain architecture via limited proteolysis [42, 43], and Ub‐ProT, which utilises Trypsin‐Resistant TUBE (TR‐TUBE) to protect ubiquitin chains from digestion [44]. These techniques give researchers the unique ability to investigate the ubiquitin code during different conditions.

Alternatively, isotopically labelled peptides corresponding to diGly‐modified residues in the ubiquitin protein sequence can be introduced to a biological sample as a reference during mass spectrometry quantification. The inclusion of these synthetic peptides enables the absolute quantification of all ubiquitin chain‐linkages in a sample: an approach termed ‘Ub‐AQUA’ [45]. Rather than just a fold change, Ub‐AQUA provides the stoichiometry of ubiquitin chains—a major advantage over relative quantification. Interestingly, the Ub‐AQUA technique has unveiled that, in skeletal muscle obtained from 8‐ to 12‐week‐old mice, 8.7% of total ubiquitin is formed by polyubiquitin chains and over half of these are K33‐linked [46]. The high stoichiometry of K33‐linked polyubiquitin chains was a distinct feature of muscle, as most other mouse tissues were dominated by K48‐linked polyubiquitin chains. Ubiquitin protein standard absolute quantification (Ub‐PSAQ) is another method for absolute quantification of ubiquitin [47]. Ub‐PSAQ uses isotopically labelled protein standards corresponding to free ubiquitin and ubiquitin conjugates which are added to lysates before digestion. By including affinity reagents selective for capturing specific polyubiquitin chains, Ub‐PSAQ is capable of providing absolute quantification of ubiquitin chains.

## 4 Applications of Ubiquitylomics

Ubiquitylomics is a tool that can be employed to investigate different biological questions. Most commonly, ubiquitylomics is employed to uncover signalling networks and protein interactions but can also be used for drug discovery and biomarker identification, showcasing its effectiveness in both experimental and clinical settings.

### 4.1 Analysing the Dynamics of Ubiquitylation

Ubiquitylated proteins often have a short life‐expectancy as many are degraded by the proteasome. Proteasome inhibition enables the capture of ubiquitylated proteins destined for proteasomal degradation, thereby capturing highly dynamic ubiquitylated proteins. As a result, proteasome inhibition can increase the detection of ubiquitylated peptides two to three fold following K‐ε‐GG antibody enrichment [48]. Of note, proteasome inhibition induces proteotoxic stress which creates additional ubiquitylated substrates for protein quality control. To distinguish dynamic and protein quality control ubiquitylation, one ubiquitylomics study employed an 8‐h time‐course with the proteasome inhibitor bortezomib [13]. The authors reasoned that ubiquitylated proteins which increased exclusively at the later 8‐h time‐point most likely arose from proteotoxic stress rather than dynamic regulatory ubiquitylation. Of note, the ubiquitylation sites in this group outnumbered the sites which increased only at the 2‐h treatment time‐point. Therefore, longer treatments of proteasome inhibition may capture more proteotoxic ubiquitylation created by the treatment as opposed to naturally occurring dynamic ubiquitylation.

### 4.2 Capturing Substrates of Ubiquitin‐Regulating Enzymes

Mapping an E3 ligase or DUB to specific ubiquitylation sites on a substrate remains the holy grail in ubiquitylation research. Modern mass spectrometers are well equipped to identify whether a substrate has become ubiquitylated or deubiquitylated; but identifying the enzymes responsible presents a greater challenge. This is mainly because ubiquitylation can be regulated by approximately 600 E3 ligases and 100 DUBs in humans [9], with partially overlapping substrates and varying degrees of redundancy.

Studies have utilised chemical or genetic modification methods to heighten or suppress the expression or activity of an E3 ligase or DUB, followed by subsequent ubiquitylomics. Proteins that undergo significant changes in their ubiquitylation status following the altered expression/activity of an E3 ligase or DUB can be deemed as potential substrates. This approach has been used to study the substrates of the largest family of E3 ligases called the Cullin‐RING E3 ligases [49]. Chemical inhibition of this E3 ligase family coupled with ubiquitylomics identified 410 candidate substrates, with 108 also displaying altered stability. Potential substrates of E3 ligases implicated in muscle atrophy have also been identified using a similar approach. For example, the overexpression of MuRF1, ASB2β and mutant KHL40 in mouse or zebrafish coupled with ubiquitylomics revealed potential substrates of these E3 ligases in skeletal muscle [50, 51, 52]. To the best of our knowledge, identification of DUB substrates using ubiquitylomics has yet to be performed in skeletal muscle. In the case of cellular models, genetic manipulation of DUBs coupled with ubiquitylomics analysis has been conducted to identify USP30 substrates, demonstrating that USP30 targets multiple mitochondrial proteins involved in the regulation of mitophagy [53]. Since then, the same approach has been used to study many other DUB substrates, contributing to the establishment of a database of DUB substrates [54].

Other approaches attempt to ‘capture’ E3 ligase or DUB substrates through protein–protein interactions. Affinity‐based capture such as co‐immunoprecipitation are often used to capture protein–protein interactions; however, a major disadvantage is the difficulty in identifying transient and low‐affinity interactions. To overcome these pitfalls, synthetic fusion proteins can be expressed in cells to ‘trap’ protein interactions. E3 ligase–substrate interactions can be captured through fusion of ubiquitin to the E3 ligase, a reagent known as Ubiquitin‐Activated Interaction Traps (UBAITs) [55]. Substrate trapping can also be performed through exogenous co‐expression of an E3 ligase fused with a TUBE [56]. TUBEs bind to polyubiquitylated substrates in cells, protecting them from DUBs and proteasome‐mediated degradation. Afterwards, immunoprecipitation (often targeting the tag fused to the TUBE e.g. anti‐FLAG) can be used to selectively enrich ubiquitylated substrates prior to mass spectrometry. TR‐TUBE offers an additional advantage by protecting both the TUBE and ubiquitin from trypsin digestion [57]. This is beneficial as digested peptides from TUBE and ubiquitin can introduce a substantial source of background noise during mass spectrometry analysis.

Proximity labelling enzymes, such as APEX2, BioID and TurboID, have also been successfully deployed for capturing putative E3 ligase or DUB substrates in cells. When fused to an E3 ligase or DUB, proximity labelling enzymes typically work by labelling nearby proteins with biotin in live cells. Biotinylated proteins (representing putative substrates) can then be enriched through streptavidin affinity pull‐down and analysed using mass spectrometry. The development of more catalytically efficient proximity labelling enzymes means it is now possible to label interacting proteins within a few minutes, enabling capture of transient signalling responses [58]. It should be noted, however, that this method will also label non‐substrates that come into proximity with the E3 ligase or DUB of interest and so further validation steps (such as in vitro ubiquitylation/deubiquitylation assays) should be performed to confirm authenticity. To improve specificity for capturing ubiquitylated substrates and mitigate non‐substrate capture, recent studies have implemented additional modifications to these proximity labelling enzymes, for example, BioE3, E‐STUB and Ub‐POD [59, 60, 61].

### 4.3 Targeted Protein Degradation

Ubiquitylomics has also been used to detect substrates of E3 ligases in the context of targeted protein degradation. Small molecules such as proteolysis targeting chimera (PROTAC) and molecular glues have been developed to redirect E3 ligases for the degradation of non‐native substrates (known as neo‐substrates) associated with disease [62]. However, one concern regarding the effectiveness of PROTACs and molecular glues as therapeutic agents is how selective the redirected E3 ligase will be to its target neo‐substrate. Any off‐target ubiquitylation could result in the unwanted degradation of physiologically important proteins. Ubiquitylomics has been used to profile protein ubiquitylation following targeted protein degradation to determine the specificity of PROTACs/molecular glues. To give a classic example, quantitative ubiquitylomics analysis revealed that Lenalidomide—a drug used to treat multiple myeloma—works as a molecular glue by selectively ubiquitylating and degrading the transcription factors IKZF1 and IKZF3 [63].

### 4.4 PTM Crosstalk

The proteome‐wide crosstalk between ubiquitylation and other PTMs can be explored using mass spectrometry. Interplay between different PTMs on a single protein, termed PTM crosstalk, adds an additional layer of complexity to cellular regulation. Beyond the impact of individual PTMs, their inter‐regulation influences signalling pathways, contributing to the intricate maintenance of cellular homeostasis. PTM crosstalk can be assessed by mass spectrometry through additional steps such as serial enrichment and deep fractionation [64]. Serial enrichment is a popular approach for analysing multiple PTMs in the same sample, utilising the flow‐through of one PTM enrichment for subsequent enrichment of another PTM [65, 66]. Whilst serial enrichment allows for smaller sample input, this approach can result in the loss of PTM information. In one comparison, prior enrichment of phosphorylated peptides reduced the detection of ubiquitylated peptides by 13% when compared to isolated enrichments [65]. Although some of these losses could be a result of the additional handling steps required, it is also likely that other losses occur due to both PTMs co‐existing on the same peptide, which is captured in the initial enrichment and thus absent from subsequent enrichments. Therefore, consideration should be taken over the most appropriate enrichment order to reduce loss of PTM information, for example, perhaps prioritising the least abundant PTM. At the instrument level, coupling mass spectrometry with high‐field asymmetric ion mobility spectrometry (FAIMS) represents a technological advance that uses multiple forms of online peptide separation to improve the dynamic range of detection for less abundant PTM‐containing peptides [67].

The application of mass spectrometry has substantially improved our understanding of the occurrence and functional importance of PTM crosstalk with ubiquitylation. For example, one study investigated the directionality of PTM crosstalk, identifying conserved phosphorylated sites which precede ubiquitylation [68]. This crosstalk directionality occurs with phosphodegrons, in which protein phosphorylation is recognised by an E3 ligase, leading to subsequent ubiquitylation and protein degradation. Phosphorylation can also regulate substrate ubiquitylation independent of protein degradation, for example during DNA repair [69]. On the other hand, there are examples of phosphorylation preventing protein ubiquitylation [70]. Beyond phosphorylation, there are many other PTMs that display crosstalk with ubiquitylation, highlighted more comprehensively in another review [71]. One example worth noting is the crosstalk between ubiquitylation and acetylation—both occurring on lysine residues. A lysine residue harbouring an acetyl modification may prevent ubiquitin attachment at this position. By comparing ubiquitylome and acetylome datasets obtained from human cell lines, it was revealed that 30% of acetylated lysines can also be modified by ubiquitin [23]. In patient‐derived tumour tissue, a subset of lysine sites were inversely modified by ubiquitylation and acetylation, suggesting these two modifications work in an antagonistic manner [37]. In agreement with this finding, acetylation at both lysine and N‐terminal residues has been reported to protect proteins from ubiquitin‐mediated degradation [72, 73]. Lysine acetylation also occurs on ubiquitin itself, which can inhibit polyubiquitin chain assembly, thus impacting chain architecture [74]. These studies demonstrate how different PTMs communicate to alter ubiquitin signalling. Therefore, coupling ubiquitylomics with other PTM‐enriched proteomics will help identify ‘switches’ of ubiquitylation that regulate health and disease, offering targets for therapeutic intervention.

## 5 Ubiquitylomics in Skeletal Muscle

**FIGURE 3:**
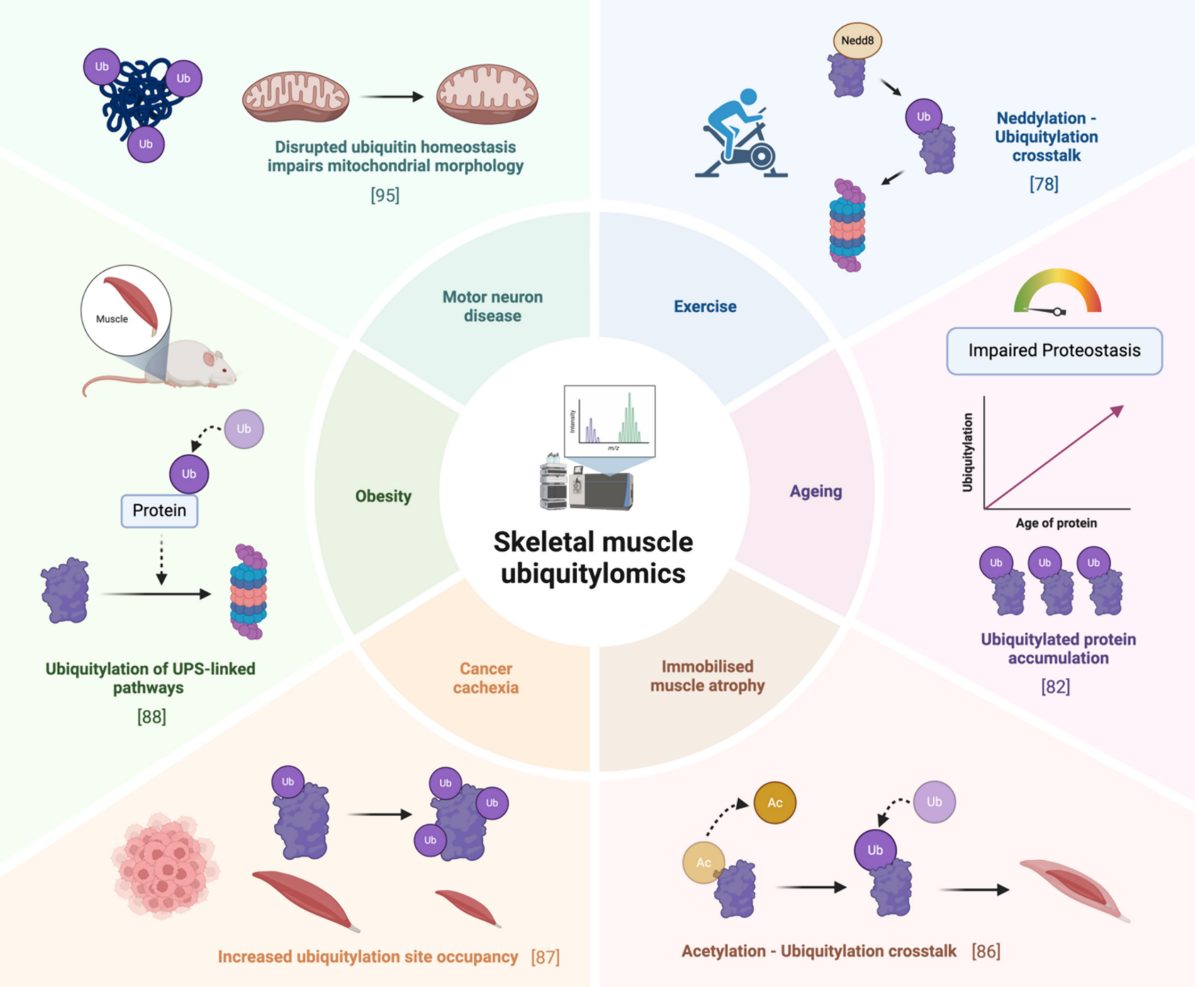
Schematic displaying the results of skeletal muscle ubiquitylomics experiments in different physiological or pathological states.

Ubiquitylomics is evidently a powerful tool for studying protein ubiquitylation, with continuous advancements in methodological approaches providing new ways to unravel the complexities of ubiquitylation in cellular biology. In skeletal muscle, ubiquitylomics is commonly framed in the context of change induced by various forms of perturbation, including pathological, physiological or interventional (Figure 3).

### 5.1 Exercise

Exercise undoubtedly stands out as the most well‐established method for maintaining skeletal muscle health, known to induce many signalling pathways in response to changes in the cellular environment. Exercise attenuates the degenerative ageing hallmarks [75] and can slow down the progression of skeletal muscle atrophy through improving protein quality control [76]. In skeletal muscle, strenuous exercise activates the Ubiquitin–Proteasome System (UPS), presumably to remove damaged proteins [77]. To understand the potential mechanisms responsible for this increase, one study employed diGly peptide enrichment to investigate the effect of an acute bout of intense aerobic exercise on the skeletal muscle ubiquitylome [78]. GSEA revealed that several pathways, including muscle contraction and glycolysis, displayed altered protein ubiquitylation during exercise. Future work is required to determine whether ubiquitylation of these proteins regulates exercise‐induced improvements in muscle mass and energy metabolism. Rapid alterations in protein ubiquitylation were found alongside increases in NEDDylation, leading the authors to hypothesise that crosstalk between these two PTMs is involved in UPS activation. Changes in ubiquitylated proteins were not seen 2 h after exercise, highlighting the dynamic and transient nature of protein ubiquitylation. This is in stark contrast to the phospho‐proteome, in which nearly 3000 phospho‐sites were differentially regulated even 3 h after intense sprint exercise [79]. To date, there is no study looking into the ubiquitylome following resistance or endurance exercise; given the utilisation of different metabolic and contractile proteins during sprint, endurance and resistance exercise, one might expect unique changes in protein ubiquitylation with each exercise bout.

### 5.2 Ageing

During ageing, there is a decline in protein quality control, resulting in an accumulation of protein aggregates and a global loss of proteostasis—a well‐established hallmark of ageing also seen in skeletal muscle [80, 81]. Given the role of ubiquitylation in regulating protein quality control, ubiquitylomics has been employed to identify ubiquitin‐mediated impaired proteostasis in aged skeletal muscle. When combined with stable isotope labelling, ubiquitylomics analysis revealed an age‐related increase of long‐lived ubiquitylated proteins in *Drosophila* muscle [82]. To our knowledge, this method has not been applied in mammalian skeletal muscle tissue; however, similar results were observed in mouse liver tissue [83]. These long‐lived ubiquitylated proteins could represent protein aggregates that are ubiquitylated but not efficiently degraded in skeletal muscle. The relationship between protein aggregates and ageing in skeletal muscle has not been extensively studied. Nevertheless, mass spectrometry has revealed tissue‐specific aggregates in aged African killifish *N. furzeri* and found DHTKD1, a mitochondrial enzyme involved in the degradation of several amino acids, is aggregation‐prone in skeletal muscle [84]. Performing ubiquitylomics analysis on insoluble aggregates would enable the identification of ubiquitylated proteins that contribute towards age‐related impaired muscle proteostasis.

### 5.3 Muscle Atrophy and Disease

Protein ubiquitylation has long been known to contribute towards muscle atrophy, promoting myofibrillar protein degradation through the UPS. Aligning with this concept, ubiquitylomics studies conducted in denervated‐ and immobilised‐induced muscle atrophy show global increases in myofibrillar protein ubiquitylation [85, 86]. By integrating these findings with additional data obtained by mass spectrometry, both studies were able to provide novel mechanistic insights. For example, actin and myosin heavy/light chain became ubiquitylated following immobilisation and were also deacetylated [86]. Therefore, the antagonistic crosstalk between acetylation and ubiquitylation appears to be involved in immobilised‐induced muscle atrophy. Furthermore, denervation altered the expression of 105 proteins associated with ubiquitylation, including the upregulation of E3 ligase TRIM25, which had not previously been associated with muscle atrophy [85]. By obtaining large datasets for both total and ubiquitylated proteins, this study offers a valuable resource to study potential targets of ubiquitin‐associated enzymes during denervation‐induced atrophy.

Tumour‐bearing mice display increasing protein ubiquitylation as they experience muscle atrophy (cancer cachexia), suggesting that ubiquitylation correlates with the reduction in muscle mass [87]. Interestingly, the authors were able to show that ubiquitylation increased most on additional sites of proteins already ubiquitylated before muscle atrophy occurred. Therefore, during cancer, proteins ubiquitylated at early stages may be targets for additional ubiquitylation during muscle atrophy. Most of these sites were dependent upon the E3 ligase MuRF1, and GSEA revealed they belonged to proteins involved in muscle contraction, cytoskeleton, sarcoplasmic reticulum and glycolysis.

As a highly metabolic tissue, skeletal muscle undergoes changes in response to metabolic health. Obesity causes a deterioration in metabolic health which compromises the function of skeletal muscle, e.g. insulin resistance. Based on the observation that the activity of the UPS is greater following a high‐fat diet, one group investigated the effects of this change on the mouse skeletal muscle ubiquitylome [88]. They found that, whilst the total number of ubiquitylated proteins and ubiquitylated sites remained largely unchanged, individual protein ubiquitylation changes were evident, including those involved in proteasome‐mediated degradation. This emphasises the importance of employing ubiquitylomics to identify such individual differences in an unbiased way. This work made parallels between UPS activation and energy metabolism in obesity by utilising a multi‐omics approach. By combining RNAseq, proteomics, ubiquitylomics and metabolomics, they found the UPS activator Nfe2l1, which promotes degradation of K48‐linked ubiquitylated proteins, encourages glycolytic metabolism in fast‐twitch muscle fibres. These findings illustrate how protein ubiquitylation can shape the landscape of energy metabolism in skeletal muscle.

Loss of motor neuron function can also contribute towards skeletal muscle atrophy. Disrupted ubiquitin signalling and subsequent loss of proteostasis is a feature of many neuromuscular diseases [89]. For instance, lack of the DUB Uchl1 in Gracile Axonal Dystrophy (GAD) impairs the synaptic transmission at neuromuscular junctions, loss of function in the E3 ligase coding gene Ube3a disrupts proteasome activity in Angelman Syndrome (AS), the E3 ligase Rnf126 degrades frataxin which is reduced in Friedreich Ataxia (FRDA), reduced levels of the E1 ubiquitin‐activating enzyme Uba1 disrupts myelin protein expression in Schwann cells isolated from Spinal Muscular Atrophy (SMA) mice and the accumulation of ubiquitylated protein inclusions in neuronal cells disrupts proteostasis in Amyotrophic Lateral Sclerosis (ALS) [90, 91, 92, 93, 94]. Ubiquitylomics has been employed on different cell models of ALS, identifying targets of altered ubiquitylation which may contribute towards the pathology. For instance, cells expressing misfolded superoxide dismutase 1 experienced increased ubiquitylation of mitochondrial proteins corresponding to mitochondrial defects [95]. Moreover, cells lacking functional Cyclin‐F impaired the ubiquitylation of Hsp90ab1, disrupting its chaperone capabilities [96].

## 6 Challenges of Ubiquitylomics in Skeletal Muscle

Whilst ubiquitylomics‐based experiments have identified ubiquitin signalling networks that drive biological processes in skeletal muscle, the number of these studies is substantially lower than in many cells and tissues. This is most likely due to the difficulties in achieving a deep coverage of the muscle ubiquitylome.

The number of ubiquitylated proteins identified by mass spectrometry is largely affected by the abundance distributions (or dynamic range) of proteins in the sample. Until recently, mass spectrometry‐based proteomics typically employed data‐dependent acquisition (DDA), in which only the most‐abundant peptides observed by the mass spectrometer are analysed further. Highly abundant proteins contribute to a large proportion of the total peptides injected into the mass spectrometer, supressing the detection of less abundant peptides. This problem is particularly apparent when analysing skeletal muscle tissue, which is composed of large and abundant structural and contractile proteins that make up a sizeable proportion of the total peptide content. For example, the structural protein titin is the largest protein in the human proteome, comprising ~30 000 amino acids and exceeding 3000 kDa. Therefore, peptides derived from titin are very abundant and frequently detected throughout the mass spectrometer detection period. Based on the sum of mass spectra, one study found that the 10 most abundant proteins in skeletal muscle make up half of the total protein mass in skeletal muscle, with titin accounting for 16% [97].

**FIGURE 4:**
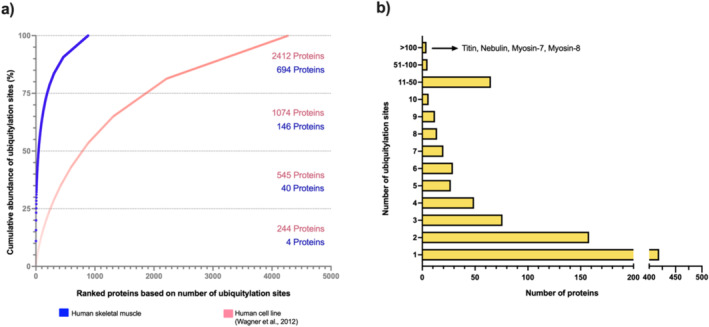
Dynamic range in the number of ubiquitylation sites detected on each protein in skeletal muscle. (a) Ubiquitylomics data from human skeletal muscle (*n* = 3) was compared against published data combined from two human cell lines (HEK293T and MV4‐11) [23]. Proteins were ranked based on the number of ubiquitylation sites detected (including only those with at least 1 site), and the cumulative abundance of ubiquitylation sites was calculated from highest to lowest rank. Each circle represents an individual protein; the number of proteins contributing to each cumulative quartile are displayed. (b) Proteins from human skeletal muscle were categorised based on the number of ubiquitylation sites detected. Each bar represents the number of proteins that contain the given number of ubiquitylation sites.

This skewed detection of a select few proteins has often prevented the deep coverage of ubiquitylated proteins. For instance, ubiquitylomics comparison across different murine tissues revealed skeletal muscle contained the lowest number of ubiquitylated sites [98]. When comparing ubiquitylomics data from human skeletal muscle and two different human cell lines [23], fewer proteins in muscle contributed to a larger proportion of the total ubiquitylation site coverage (Figure 4). Given the high abundance of ubiquitylation sites on a few structural and contractile proteins, the coverage of ubiquitylated proteins may well be lowered by less frequent observation of lower abundance sites due to limits in the dynamic range of detection. Therefore, a substantial proportion of the ubiquitylated proteome in skeletal muscle is likely undetected by mass spectrometry.

## 7 Considerations for Ubiquitylomics Analysis in Skeletal Muscle

To improve the depth of protein coverage detected by mass spectrometry, strategies have been developed to reduce sample complexity. A common method is orthogonal peptide fractionation (e.g., at high pH, or with strong cation exchange chromatography), which separates the sample into simpler mixtures, increasing the number of peptides that can be observed during DDA cycles [99]. When utilised in plasma, which like skeletal muscle contains a challenging protein dynamic range, high pH reverse‐phase fractionation improves the depth of protein coverage [100]. Notably, high pH reverse‐phase fractionation has been shown to improve the detection of ubiquitylation sites [101].

Studies have tried including additional fractionation steps to simplify the protein pool even further. In skeletal muscle, researchers have attempted to separate proteins based on their solubility prior to proteomics [102, 103, 104, 105]. Due to their large size and highly connected structure, highly abundant proteins in the myofiber are more difficult to solubilise. Centrifugal separation of insoluble proteins allows less‐abundant soluble proteins to be analysed separately from the more‐abundant insoluble proteins—increasing the total number of proteins detected. Through this approach, one study detected 1490 proteins in the soluble supernatant of mouse skeletal muscle that were not detected in whole tissue analysis [103]. More recently, nanoparticle protein interaction has been employed for separation of muscle proteins to further improve depth of coverage [106]. Together, these studies show the available methods for reducing sample complexity to enhance the coverage of proteins detected in skeletal muscle.

Given that protein ubiquitylation detection in skeletal muscle is dominated by a few highly abundant proteins (Figure 4), employing fractionation strategies promises to be especially fruitful for ubiquitylomics analysis. However, it should be noted that, more recently, the proteomics field is broadly moving away from such fractionation approaches as advances in instrument speeds and sensitivity offer greater dynamic ranges of detection. It remains to be seen if these advancements will provide similar impacts on the challenges of skeletal muscle proteomics and ubiquitylomics.

The method employed for data acquisition on the mass spectrometer also plays a crucial role in determining the depth of protein coverage. As mentioned earlier, DDA characterises a limited number of peptides based on abundance. Recently, data‐independent acquisition (DIA) has emerged as a powerful alternative to DDA. DIA fragments all peptide ions within a mass to charge window, resulting in less bias towards highly abundant proteins. As a result, DIA has been able to identify up to 70 000 ubiquitylated peptides, significantly increasing the detection limit in a single mass spectrometer run [107, 108]. DIA will likely improve the number of ubiquitylated peptides/proteins detected in skeletal muscle, combating the underrepresentation of less‐abundant proteins often seen in DDA.

## 8 Conclusions

**FIGURE 5:**
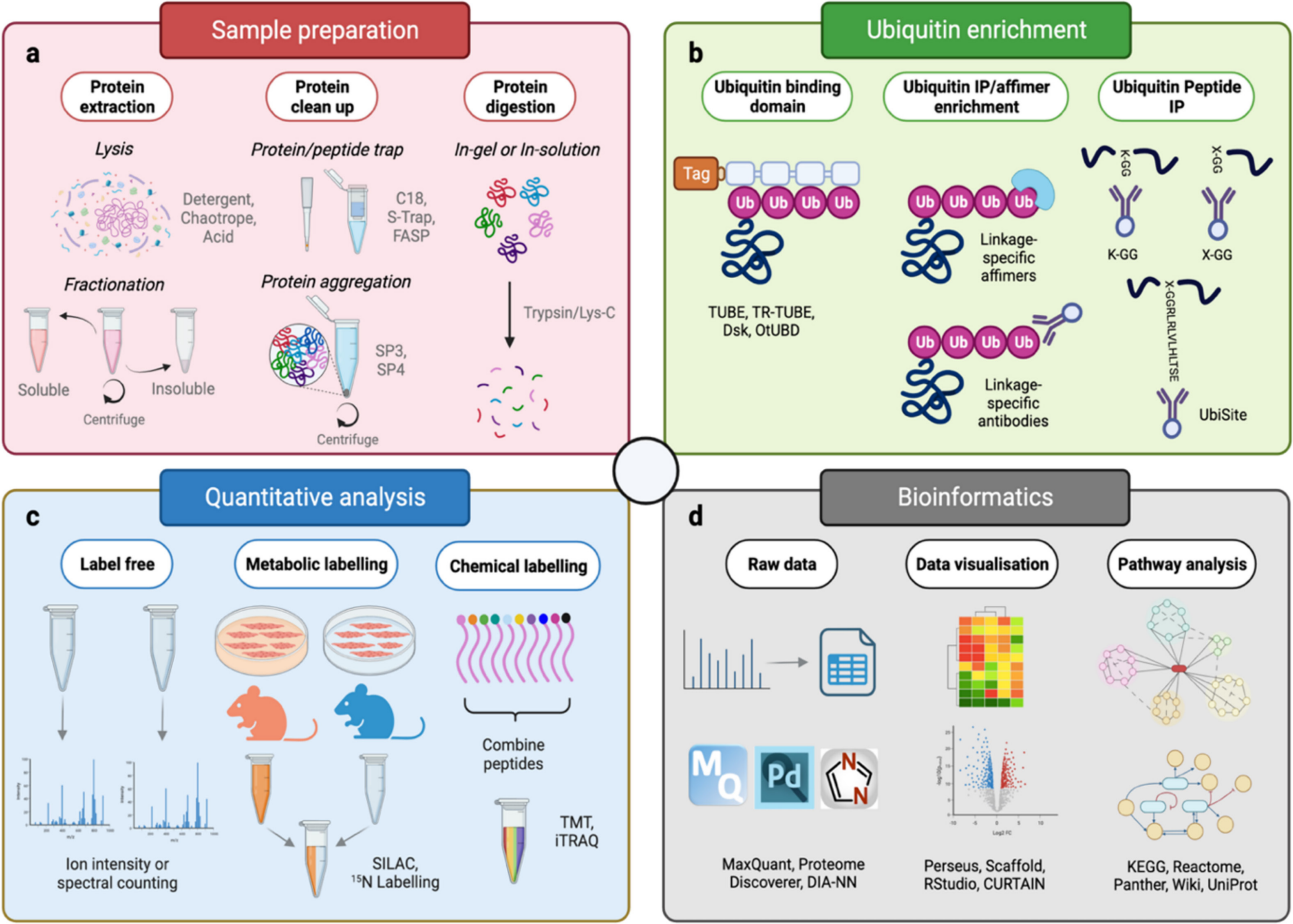
Schematic displaying the key steps when performing mass spectrometry‐based ubiquitylomics experiments. (a) Sample preparation is used to ensure sample compatibility with proteolysis, liquid chromatography and mass spectrometry. Protein extraction and solubilisation requires the use of harsh chemical agents, for example, detergents and chaotropes, that can interfere with typical bottom‐up proteomics workflows. Various clean‐up methods are available for isolating proteins or peptides, for example, protein aggregation or trapping, allowing the removal of contaminants. (b) Ubiquitin enrichment is used to deplete non‐ubiquitylated peptides/proteins that would interfere with detection. Multiple tools are available for ubiquitin enrichment at both the protein and peptide level, such as ubiquitin binding domains, antibodies and affimers, each with different advantages and disadvantages. (c) Quantitative analysis is performed to identify relative abundance differences in ubiquitylated peptides/proteins between samples. The label‐free approach compares either ion intensities or spectral number of a given protein. Data‐independent acquisition (DIA) is mostly limited to label‐free quantification, whilst data‐dependent acquisition (DDA) allows for label‐based quantification and multiplexing. Metabolic labelling involves the use of ‘heavy’ and ‘light’ isotopes, which are incorporated into proteins (e.g., in lysine and arginine residues for SILAC), and relative quantification is performed by comparing the isotope intensities of a given protein's peptides. Chemical labelling modifies all peptides covalently with isobaric tags, conferring identical chemical properties, but reporter ions differentiated by isotopic distributions. The intensities of the reporter ions (which are cleaved off during MS fragmentation) is used to infer relative quantification of a given protein. (d) Bioinformatics is required for the deconvolution of spectral data obtained from the mass spectrometer. Various software is available which deal with raw MS data and employ database searching and filtering for the identification and quantification of peptides/proteins and their modifications, for example, diGly for ubiquitylation. Biologically meaningful data can then be visualised through different displays, for example, volcano plots and heatmaps, using programs that often include statistical testing. Pathway analysis can also be employed to search for biological pathways driven by differentially regulated ubiquitylated proteins.

With the trajectory of methodological advancements, the analysis of thousands of ubiquitylated peptides in skeletal muscle has become feasible. Therefore, it may be of interest for researchers to become familiarised with the technical and analytical steps involved (Figure 5). More detailed information on these steps is covered in other reviews [109, 110, 111, 112]. One major challenge of ubiquitylomics is to understand how each ubiquitylation site integrates into the biological system. Integrating this method into a multi‐omics framework for parallel analysis of nucleic acids, proteins and metabolites could help establish mechanistic insight into the order of molecular events. The next phase of ubiquitylomics could also uncover the spatial and temporal regulation of skeletal muscle ubiquitylation, harnessing technologies developed in other ‐omics fields, for example, stable isotope tracers for flux analysis, and mass spectrometry imaging for spatial resolution [113, 114]. User‐friendly web‐based computational tools, such as MSstatsShiny, WebGestalt and CURTAIN, make ubiquitylomics data analysis far more accessible to muscle physiologists and exercise scientists with limited bioinformatics experience [115, 116, 117]. Even if not directly engaged in ubiquitylomics‐based experiments, researchers should make use of the rich datasets provided by ubiquitylomics studies, deposited in accessible databases such as PRIDE [118]. Searching through ubiquitylomics datasets to determine whether specific proteins in skeletal muscle are ubiquitylated in certain conditions is likely to prove beneficial in both clinical and experimental settings.

## Ethics Statement

The authors comply with the Ethical guidelines for authorship and publishing in the *Journal of Cachexia, Sarcopenia and Muscle*. Ethical approval for human muscle research was obtained through the East Midlands–Derby Research Ethics Committee (18/EM/0004), conformed to the requirements of Research Governance at the University of Birmingham and was conducted in accordance with the Declaration of Helsinki.

## Conflicts of Interest

The authors declare no conflicts of interest.
